# Cytochrome modulation decreases the risk of cataract: Is the basic hypothesis flawless?

**DOI:** 10.4103/0253-7613.58516

**Published:** 2009-10

**Authors:** Rajiv Mahajan, Kapil Gupta

**Affiliations:** Department of Pharmacology, Biochemistry, Adesh Institute of Medical Sciences & Research, Bathinda - 151 109, Punjab, India. E-mail: drrajivmahajan01@yahoo.co.in

Sir,

We have read the article, “Is cytochrome modulation the new frontier for decreasing the risk of cataract?” published in *IJP*, April 2009[1] with great enthusiasm. The study is based on the hypothesis  excess glucose is converted to sorbitol (which can cause cataract) by enzyme aldose reductase using NADPH as a cofactor, and electron transfer from NADPH further depends on the cytochrome P450 system. Thus, it was postulated that by inducing or inhibiting cytochrome one can alter the activity of aldose reductase, formation of sorbitol, and the occurrence of cataract. We feel that this hypothesis is not flawless and would wish to make the following observations in this regard:

1. NADPH is used as a cofactor for aldose reductase and it is also used to transfer electrons in the cytochrome P450 system which then participates in the metabolism of drug/endogenous metabolites.[2] Thus, any reaction that will alter the synthesis of NADPH will only be able to affect the aldose reductase pathway. But, NADPH is biosynthesized by the hexose monophosphate shunt (HMP shunt),[3] (and not by the cytochrome system) and so by modulating cytochrome activity one cannot alter the activity of aldose reductase.
2. The aldose reductase pathway is absent in liver.[3] It therefore appears that the very site where induction of enzymes occur maximally lacks the sorbitol pathway for cytochrome modulation to be able to affect the cataract formation.
3. It has been hypothesized that by simple alteration in the medication (CYP inhibitors instead of CYP inducers) one can reduce the risk of cataract. If we assume that cytochrome modulations alter the activity of aldose reductase through NADPH, then this would mean that enzyme induction will decrease the amount of NADPH which is available to be used as a cofactor by aldose reductase (because more NADPH is now being used in cytochrome pathway due to its induction) and thus decrease the rate of this enzymatic reaction. In other words, it appears that enzyme inducers will decrease the amount of sorbitol formed and vice-versa.
4. If we assume that drugs directly modulate the activity of aldose reductase, then one has to keep in mind that as stated earlier, aldose reductase is absent in liver, where most of diltiazem (about 60%) undergoes first pass metabolism.[4] Direct involvement of drugs with aldose reductase at this site is out of question. Although, besides lens, aldose reductase is present at other sites like kidney, placenta, and seminal vesicles,[3] its presence at these places cannot be responsible for cataract formation. Moreover, the drugs used in this experiment, i.e. pioglitazone and diltiazem, are bound to plasma proteins to an extent of 99% and 80%, respectively.[4] So, it is doubtful if they will accumulate in eye in sufficient quantities to have their effect.
5. The authors have used pioglitazone as an enzyme inducer but doubts have already been raised on its potential to induce CYP3A4.[5,6]
6. Authors have used galactose-rich diet to induce cataract although it has been argued that galactose fed rats are not a good model of diabetes and the subsequent induction of cataract.[7] It has also been pointed out that the use of galactose fed animal models leads to subsequent clinical failure of aldose reductase inhibitors.[7]
